# Making Machine Learning Accessible for Developmental Science: The Case of Automated Face Detection

**DOI:** 10.1111/desc.70148

**Published:** 2026-04-20

**Authors:** Teodor Y. Nikolov, Julia Yurkovic‐Harding, Tamas Foldes, Jessica Bradshaw, Yu‐Kun Lai, Hana D'Souza

**Affiliations:** ^1^ Centre for Human Developmental Science School of Psychology Cardiff University Cardiff UK; ^2^ Department of Psychology University of South Carolina Columbia South Carolina USA; ^3^ Carolina Autism and Neurodevelopment Research Center University of South Carolina Columbia South Carolina USA; ^4^ Oxford Internet Institute University of Oxford Oxford UK; ^5^ School of Computer Science and Informatics Cardiff University Cardiff UK

**Keywords:** egocentric vision, face detection algorithms, head‐mounted eye‐tracking / camera (headcam), infants, machine learning, toddlers

## Abstract

The last decade has seen rapid advancements in machine learning, significantly transforming fields like cybersecurity and healthcare. Developmental science has been slower to adopt these technologies. Yet, machine learning holds immense potential to transform this field, enabling scalable and data‐driven insights into developmental processes. Broader adoption is currently hindered by challenges in *algorithm selection* and *technical implementation*. We address these barriers by focusing on an area that has reached high sophistication from a machine learning perspective while also being of significant interest to developmental scientists: *face detection*. Face detection is crucial for analysing visual experiences through children's dynamic, first‐person views. Automatising this process allows efficient handling of large egocentric datasets, enabling well‐powered studies otherwise limited by labour‐intensive manual annotation. Here, we systematically evaluated 13 state‐of‐the‐art face detection algorithms (DeepFace library) using data from two increasingly common developmental methodologies involving children under 3 years of age: *head‐mounted eye‐tracking* in more structured settings (*N* = 20; *n* = 10 4‐month‐olds, *n* = 10 8‐month‐olds) and *head‐mounted cameras* in naturalistic home environments (*N* = 10 18–29‐month‐olds). Benchmarking these algorithms against manual annotations revealed that *YOLOv11Face (M)* and *RetinaFace* consistently outperformed others in terms of precision and recall, exhibiting strong concordance with manual ratings, lower error, reduced systematic deviation and robust rank‐order correlations with manual annotations. To facilitate broader adoption, we introduce an accessible face detection tool (*TinyExplorer Detection App*), promoting efficiency, scalability, and innovation in developmental science by widening access to machine learning.

## Summary

- Machine learning advancements offer significant potential for developmental science but are currently largely unvalidated and underutilised in this field.
- Automated face detection is transformational for analysing infants’ visual experiences in dynamic, first‐person views, significantly reducing manual annotation effort.
- Thirteen state‐of‐the‐art face detection algorithms were systematically evaluated for their suitability in developmental research, using head‐mounted eye‐tracking and camera data from children under 3 years of age.
- We introduce an accessible, user‐friendly face detection app designed to promote the widespread adoption of machine learning, enhancing scalability and innovation in developmental science.

## 1 Background

Recent years have seen exponential advancements in machine learning, with algorithms reaching unprecedented levels of sophistication. As a branch of artificial intelligence, machine learning detects patterns in data and generalises from examples to improve predictions, reducing reliance on explicitly programmed rules or instructions. These developments have transformed various fields, including cybersecurity (Handa et al. 2019) and healthcare (Waring et al. 2020). However, developmental science has been slower to adopt these technologies (cf. e.g., Gilkerson et al. 2017; Lavechin et al. 2020; Long, Kachergis, et al. 2022; Long, Sanchez, et al. 2022; Nikolov and D'Souza 2026; Räsänen et al. 2021). Yet, machine learning holds immense potential to transform the developmental science field by providing scalable, automated annotation methods that can generate new insights into developmental processes.

We identify two key barriers hindering broader adoption of machine learning for automated annotation in developmental science: (1) Navigating the rapidly evolving field of machine learning to identify suitable algorithms for developmental research; (2) Technically implementing the computational solutions, with an emphasis on data privacy. Here, we demonstrate how these two barriers can be addressed, widening access to machine learning in the developmental science community. We specifically focus on an area that has reached high sophistication and utility from a machine learning perspective while also being of significant interest to developmental scientists: *face detection*.

Faces are fundamental to human development, contributing to critical processes such as emotion recognition, cognitive development, and social learning (for a review, see Grossmann and Johnson 2007). Early face‐to‐face interactions with caregivers play a crucial role in the development of social communication in infants, particularly within the first 6 months of life. From infancy, humans preferentially attend to faces and recognise facial expressions, a bias that facilitates crucial cognitive and social skills throughout early development (Frank et al. 2009; Johnson 2005; Kuchuk et al. 1986; Nelson 2001). By 2 months of age, infants are able to responsively smile at their parents (Anisfeld 1982; Lavelli and Fogel 2013; Malatesta et al. 1989). At 3 months of age, infants have reached the peak of eye contact with a parent (Lohaus et al. 2001). Between 4 and 8 months, infants begin to focus on their parents’ mouths, likely to support the onset of babbling (Lewkowicz and Hansen‐Tift 2012). This early attention to faces is not only critical for social communication development but also positively predicts future social communication outcomes (Salley et al. 2016). After 6 months, infants begin to initiate social interactions by looking to their caregiver's face (Cohn and Tronick 1987; Kasari et al. 1990; Messinger and Fogel 1998). This behaviour marks the foundation of joint attention, a skill that is largely implicated in later social communicative development (Adamson and Bakeman 1985; Bakeman and Adamson 1984; Bates et al. 1979; Elison et al. 2013; Striano and Bertin 2005). Face processing abilities are related to a child's joint attention abilities (Mundy 2016), underpinning theories positing that inattention to faces may have cascading effects on subsequent social communicative and cognitive development (Luotola et al. 2025; Phillips et al. 1992; Senju and Johnson 2009; Swanson and Siller 2013; Tsang et al. 2019).

Given its importance for developmental outcomes, visual availability of faces (i.e., the extent to which faces are present and visible within the child's visual scenes) has been extensively studied in developmental science. Until recently, it was typically examined in highly structured settings, including face‐to‐face assessments that are specifically designed to elicit attention to faces (e.g., Early Social Communication Scales; Mundy et al. 2003) and screen‐based eye‐tracking studies that prominently display face stimuli (e.g., watching videos of actresses acting as caregivers). In fact, 40%–80% of visual attention is allocated to faces during social video watching in screen‐based eye‐tracking studies (Hosozawa et al. 2012; Klin et al. 2002; Nakano et al. 2010). Significant technological advances have more recently allowed researchers to study the visual availability of faces in the dynamic, real‐world settings in which social interactions naturally occur, capturing children's egocentric views using *head‐mounted eye‐tracking* (HMET) and *head‐mounted cameras* (headcams). Unlike third‐person perspectives, which provide an external viewpoint, egocentric methods capture a child's visual experience from their own perspective, offering a direct and continuous record of their interactions. By preserving the natural flow of real‐world experiences, these methods provide high‐resolution data on how children engage with faces and other social stimuli in dynamic environments. This approach minimises observer interference and enhances ecological validity, allowing researchers to capture first‐person visual experiences as they unfold in everyday contexts.

HMET records the child's gaze to understand how it is dynamically allocated within their egocentric perspective (Fu et al. 2024; Slone et al. 2018). HMET is typically researcher‐administered and is often used to capture minutes‐long interactions between a child and their parent. Using HMET, the studies find that only 2%–12% of visual attention is directed towards faces (Franchak et al. 2017; Yu and Smith 2013; Yurkovic‐Harding et al. 2022; Yurkovic‐Harding and Bradshaw 2024). The number of toys available to infants and the distance from the parents are factors that influence attention to faces (Jones et al. 2017; Yamamoto et al. 2020). When infants are free to move around, postural constraints and task demands may also alter face looking (Franchak et al. 2017, 2011; Yamamoto et al. 2019).

Compared to HMET, headcams (Borjon et al. 2018) capture visual scenes, not gaze, but offer greater flexibility in multiple aspects. While HMET is typically researcher‐administered in structured settings, often requiring specialised calibration and brief, structured interactions, headcams can be worn for extended periods, allowing for continuous, naturalistic recording of a child's visual environment. This flexibility means that data collection is not restricted to laboratory settings or specific testing sessions but can take place across a range of activities in everyday environments such as the home (Jayaraman et al. 2013, 2015, 2017; Jayaraman and Smith 2019; Long, Kachergis, et al. 2022; Nikolov and D'Souza 2026; Sullivan et al. 2021), providing a richer, ecologically valid perspective on what infants and children experience. Additionally, headcams can be used across a wider range of participants, including younger infants and neurodivergent infants who may struggle with the procedures required for eye‐tracking technology. As a result, headcams are a valuable tool for capturing extended periods of egocentric visual experience, supporting research on natural interactions in early development.

Headcam studies of face availability typically capture hours‐long recordings of the infant in their home environment. These studies indicate that face availability in infants’ everyday environments varies systematically with age, with early infancy often characterised by higher face availability (approximately 25%–35%) and later developmental periods showing marked, and in some cases nonlinear, change (Fausey et al. 2016; Jayaraman et al. 2015, 2017; Long, Kachergis, et al. 2022; Nikolov and D'Souza 2026; Sugden et al. 2013). Together, this research underscores the importance of context in understanding when, why and how infants attend to faces in their everyday lives (D'Souza and D'Souza 2024).

Up until recently, the main way of detecting whether faces were or were not present in the infant views from HMET or headcams was manual annotation (Fausey et al. 2016; Jayaraman et al. 2015, 2017; Sugden et al. 2013). While rigorous, this is a time‐consuming process (e.g., Sugden et al. 2013), which limits the scalability of studies. The emergence of automated annotation tools now offers a new path forward, enabling high‐throughput and scalable analysis of infants’ interactions with their environments. An emerging body of work illustrates the potential of these tools to overcome longstanding methodological bottlenecks and expand the scope of developmental inquiry. For example, Long, Kachergis, et al. (2022) employed *OpenPose* (Cao et al. 2021) to detect faces and hands in longitudinal egocentric video data captured from infants, revealing developmental shifts in the nature and frequency of social cues in everyday visual environments. Furthermore, Long, Sanchez, et al. (2022) utilised the same pose estimation model to show how changes in infants’ posture and orientation alter their access to social information, suggesting that the development of motor abilities dynamically structures infants’ visual scenes. Yurkovic‐Harding and Bradshaw (2024) combined HMET with automated face and emotion detection (*RetinaFace;* Deng et al. 2020) to study parent‐infant interactions, identifying age‐related changes in infants’ visual attention toward facial features. Finally, Nikolov and D'Souza (2026) used automated face detection with *RetinaFace* on egocentric head‐mounted camera footage to reveal nonlinear, region‐specific developmental changes in the everyday availability and distribution of faces within the video frame across the first 3 years of life.

Yet despite these promising developments, the adoption of machine learning techniques in developmental science has progressed more slowly than in other domains. For example, cognitive neuroscience has incorporated machine learning extensively, using tools like support vector machines and deep neural networks to decode complex brain signals and identify patterns linked to cognition and disease (Woo et al. 2017). In experimental psychology, particularly within virtual reality research, machine learning enables real‐time analysis of behavioural, physiological and environmental data streams to study phenomena such as perception, emotion and social engagement (Pan and Hamilton 2018). Finally, fields such as robotics, biomedicine, and marketing have placed machine learning at the centre of innovation, applying it to everything from autonomous navigation (Chen et al. 2015) to radiological image classification (Esteva et al. 2017), and customer behaviour prediction (Chaudhary et al. 2021; Mathur et al. 2022).

If developmental science is to maximise the utility of large and complex datasets, manual or hybrid pipelines will quickly become a bottleneck. A shift towards robust algorithmic tools is not only necessary, it is foundational for the future of the field. Encouragingly, the infrastructure for this transition is emerging. Publicly available egocentric datasets, cross‐disciplinary collaborations and continual advancements in computer science are converging to support scalable, machine‐learning‐driven approaches to developmental research. By systematically evaluating algorithm performance and integration potential, these tools offer a promising route to high‐resolution, real‐world insights into attention, learning and interaction, unlocking opportunities for theory‐building that were previously beyond reach. To fully realise this potential, however, key obstacles must be addressed. Despite growing infrastructure and momentum, the successful integration of machine learning into developmental science still faces critical challenges that can hinder its widespread adoption.

We identified two main barriers to the broader application of machine learning methods in developmental research. First, *the rapidly evolving landscape of algorithms*, ranging from feature‐based techniques to sophisticated deep learning models, can be overwhelming for researchers with limited specialised technical expertise. Second, *implementing these algorithms in a privacy‐conscious and user‐friendly manner is nontrivial*, especially when working with sensitive data such as first‐person recordings from homes. This type of footage frequently contains identifiable individuals and private household environments, raising concerns around participant identifiability and privacy. In addition, these concerns extend to secure handling and storage of recordings, with local processing preferred over remote or shared processing to minimise data transfer and exposure risks. Addressing these challenges is essential for enabling developmental scientists to harness the benefits without needing to develop extensive computational pipelines from scratch.

In the present study, we utilised *face detection* as a case study. We employed DeepFace library (Serengil and Özpinar 2024), a Python‐based package, to guide our selection and implementation of face detection algorithms. This library was selected because it integrates multiple state‐of‐the‐art models and is easy to install compared to alternative libraries (e.g., InsightFace; Guo et al. 2021). All data processing runs locally rather than through external servers, which is particularly important for maintaining privacy when working with sensitive videos. In other words, footage never leaves the researcher's machine, thereby mitigating the risk of participant identification by third parties. Its user‐friendly design allows for the execution of complex functionalities with minimal code, streamlining the workflow. Such ease of use, combined with robust community support, is particularly advantageous in developmental research, where low technical barriers and readily available troubleshooting resources directly impact research efficiency. The DeepFace library includes both lightweight and computationally intensive models, allowing for the evaluation of different options based on computational constraints. This flexibility is critical in developmental research settings, where access to high‐performance computing resources may be limited. As most face detection models implemented in DeepFace are trained on large‐scale, externally captured datasets such as WIDER FACE (Yang et al. 2016), applying them to egocentric home recordings allows us to examine performance under a markedly different visual context.

We assessed the performance of all 13 state‐of‐the‐art automated face detection algorithms included within DeepFace (Serengil and Özpinar 2024). These represent leading approaches that are widely adopted and well validated in computer vision research. Their performance was evaluated using data from two developmental research methodologies: HMET and headcams, both collected in naturalistic home environments. We systematically compared automated detection outputs from each model against manually annotated ground truth data using the *F*_1_ score. This score balances precision (proportion of detected faces that were correct) and recall (proportion of actual faces that were successfully detected), key metrics in developmental research, where false positives or negatives can significantly affect interpretations of social interactions and attention patterns. Additionally, we tested these algorithms across multiple confidence thresholds (i.e., the minimum confidence level required for the model to classify a detection as a face) to determine optimal settings, and assessed how well the automated annotations matched manual annotations. Finally, we developed a user‐friendly face detection tool, the *TinyExplorer Detection App*, to provide a programming‐free alternative to existing tools such as DeepFace (Serengil and Özpinar 2024), facilitating the adoption of top‐performing algorithms in developmental research.

## 2 Method

### 2.1 Validation Approach

To evaluate the robustness of the state‐of‐the‐art automated face detection algorithms in the DeepFace library (Serengil and Özpinar 2024), we tested their performance on two types of egocentric datasets that are common in developmental research. The first consisted of brief, semi‐structured sessions with data captured by HMET (Study 1a, 4mo, 5 minutes per session; Study 1b, 8mo, 10 minutes per session), while the second consisted of extended, naturalistic recordings captured via headcams (Study 2, 18–29mo, around 1 hour in duration). We annotated 10 videos from each study (a total of 30 annotated datasets). To make human annotation feasible given the duration of the videos, Study 1 recordings (shorter duration) were sampled at 15 Hz, and Study 2 recordings (longer duration) were sampled at 1 Hz. Frames were extracted from each video and were then scrambled to reduce potential order effects or bias. The frames were presented to human annotators, who indicated on a frame‐by‐frame basis whether at least part of a face was visible (yes/no). The same frames were subsequently processed by all 13 automated algorithms in DeepFace using default settings (Serengil and Özpinar 2024). The DeepFace library's detectors operationalise a ‘face’ as the presence of a bounding box when sufficient facial features (e.g., eyes, nose, mouth or a facial contour) are visible. Detection criteria vary across models, as each relies on different architectural and feature‐based cues rather than a common decision rule. Some models (e.g., *RetinaFace, CenterFace*, *MTCNN*) include landmark predictions and are better able to detect partially occluded or small faces; others (e.g., *YOLOFace* variants, *SSD*) trade detection accuracy for speed by prioritising faster, single‐stage detection with reduced spatial precision. Accordingly, automated detections were validated against manual annotations using precision, recall, and F_1_ scores, with F_1_ used both to compare algorithms at their default settings and to identify stable performance plateaus across decision thresholds for the top‐performing models, alongside agreement metrics assessing concordance, error and bias.

### 2.2 Study 1

#### 2.2.1 Participants

Data were collected from 19 parent‐infant dyads when the infants were 4 months old (Study 1a) and again at 8 months of age (Study 1b). Data are missing from one dyad at the 4‐month timepoint and three dyads at the 8‐month timepoint. Considering the aim of the current study, dyads were excluded from the current analyses if an experimenter who was wearing a mask was visible in the infants’ first‐person perspective (4mo *n* = 7, 8mo *n* = 4). These datasets were excluded because ground‐truth annotations included all visible human faces, and the presence of masked experimenters would introduce a distinct category of face with reduced visible features, making detection performance harder to interpret. Masked faces are also uncommon in home‐based head‐mounted camera or eye‐tracking recordings (Hofer et al. 2021; Prasad et al. 2021).

Of the remaining 23 datasets (4mo *n* = 11, 8mo *n* = 12), a randomly selected subset of 10 sessions from each timepoint (20 sessions in total) was manually annotated to serve as a benchmark for the automated detection algorithms in this study. The downsampling was motivated by the sample size used in Study 2, which included 10 participants. Demographics for the included subjects are provided in Table 1. Infants were eligible to participate in the study if the family reported that they were born at full‐term (≥37 weeks of gestation), had no vision or hearing abnormalities, no known genetic syndromes, and were at low genetic likelihood for autism spectrum disorder (ASD). These data were collected as part of a broader study of attention during naturalistic interactions in children at either low or elevated likelihood for ASD. All procedures were approved by the University of South Carolina Institutional Review Board and families gave informed consent prior to participation.

**TABLE 1:** Demographic characteristics of Study 1 sample.

	Study 1a (HMET 4mo)	Study 1b (HMET 8mo)
Sex: Male/Female	3/7	3/7
Race:		
White	4	5
Black	3	1
Asian or Pacific Islander	1	1
More than one	2	2
Not reported	0	1
Ethnicity:		
Not Hispanic or Latino	9	7
Hispanic or Latino	1	1
Not reported	0	2
Primary caregiver highest level of education:		
High school or GED	0	0
Some college	3	3
College degree	4	2
Advanced degree	3	4
Not reported	0	1
Secondary caregiver highest level of education:		
High school or GED	2	1
Some college	3	3
College degree	3	3
Advanced degree	2	1
Not reported	0	2
Total household income:		
Less than $60,000	4	3
$60,001–$100,000	2	3
$100,001–$150,000	1	1
$150,001 or higher	3	2
Not reported	0	1

#### 2.2.2 Procedures

##### 2.2.2.1 Equipment

**FIGURE 1:**
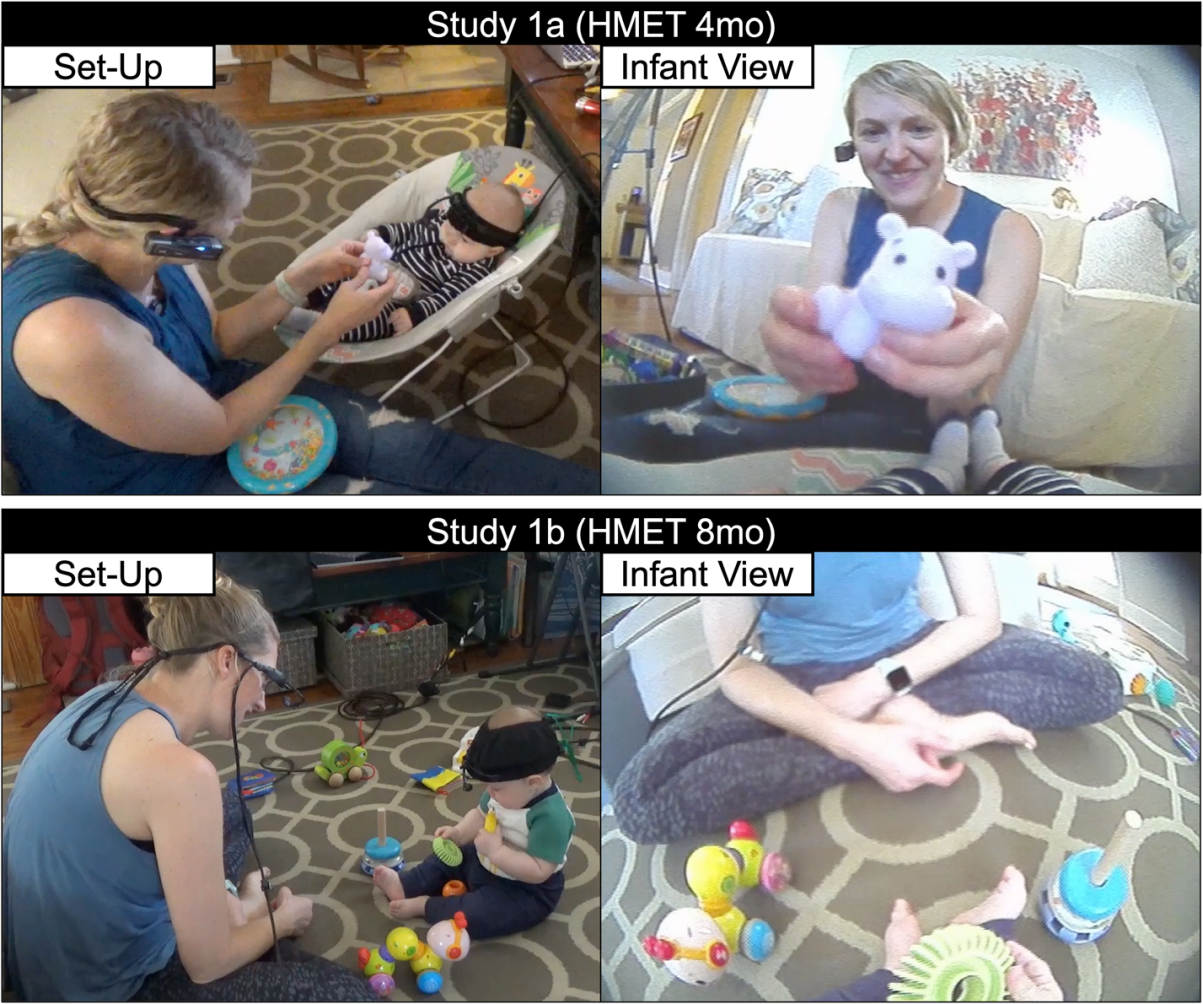
Set‐up for Study 1a (HMET 4mo) and Study 1b (HMET 8mo), with example frames shown on the right.

Infants were equipped with HMET devices (Figure 1; Positive Science, LLC) that contained a non‐intrusive infrared light pointed at the eye to capture eye movements and a scene camera on the forehead to capture the view in front of the participant at a visual angle of 90° and a resolution of 640 × 480 pixels. The infant eye tracker was affixed to a soft hat. Parents were also equipped with either a headcam (Study 1a) or a HMET (Study 1b). All interactions were recorded with an additional camera positioned at the edge of the play space. Data from all cameras were collected at ∼30 Hz, with slightly variable frame rates. Videos were resampled to 29.97 Hz following data collection to account for the variable frame rates.

##### 2.2.2.2 Video Recordings

All research visits were conducted in the family's home. Infants and parents were equipped with HMET/headcam and were provided with a standard set of age‐appropriate toys. The parents were instructed to play with their infant as they typically might at home, with no explicit instructions to play with the toys. Two experimenters were present in the home during the play interaction. The experimenters occasionally held cords from the HMET to reduce the weight on the infant's head, but otherwise stayed out of the main interaction space.

##### 2.2.2.3 Study 1a (HMET 4mo): Parent‐Infant Interaction at 4 Months of Age

Infants and parents engaged in two 5‐minute play interactions with toys. Infants were arranged in different positions for the two interactions: lying supine for one and supported in a reclined position in an infant seat or pillow that was available in the home for the other. The use of a support that was already in the infant's home ensured that the play interaction reflected a natural play interaction between the infant and parent. Infants and parents were placed directly facing each other at the start of each interaction. While parents were free to move around the play space, all stayed seated in front of their infants. One of the two toy‐play interactions was randomly selected from each dyad to serve as a benchmark for the automated detection algorithms (supine *n* = 4, reclined *n *= 6).

##### 2.2.2.4 Study 1b (HMET 8mo): Parent‐Infant Interaction at 8 Months of Age

Dyads completed two 10‐minute play interactions with toys. The set‐up and instructions for the interactions were the same, with a split introduced to enable recalibration. Both the infant and the parent were free to move about the play space as they desired. Of the 10 infants, nine were in a seated position, and one was in a prone position for most of the play session. Infants and parents typically began the interaction directly facing one another or seated at a 90° angle from each other. One of the two toy‐play interactions was randomly selected from each dyad to serve as a benchmark for the automated detection algorithms.

##### 2.2.2.5 Manual Annotation of Faces in Infant's First‐Person View

In Study 1a (HMET 4mo) and Study 1b (HMET 8mo), infants’ scene videos were downsampled to 15 Hz (15 frames per second), and images were extracted for both manual and automated face detection. After downsampling, Study 1a included a total of 43,690 frames (*Mdn* = 4,576.5, *IQR* = 133, range = 2,407–4,788). Study 1b included 58,716 frames (*Mdn* = 4,711.5, *IQR* = 852, range = 4,488–11,700). Human annotation was completed using a custom MATLAB program. Two human annotators coded the number of faces available in each video. Annotators were trained to identify a face as present if any part of the face was visible in the video frame (i.e., a single eye visible was counted as a face). Each annotator underwent a 30‐minute training session using example data, which included faces that were difficult to detect (i.e., in the background, blurry, partially occluded). The annotators were not blind to the study timepoint or participant ID number. Annotators coded every 5th frame of the video from the infant's first‐person perspective. Each annotator was assigned a different onset frame in the sampling scheme (i.e., one coded frames 1, 6, 11…; the other coded 3, 8, 13…), so they annotated different subsets of the video. These frames were presented in random order to minimise bias, and each image was presented only once. After annotation, the frames were processed sequentially. If two adjacent annotated frames contained the same number of faces, all frames between them were assumed to contain that same number, and no additional annotation was needed. If the number of faces differed between two adjacent coded frames, the frames between them required further annotation. The frames in this second round of annotating were again presented to the annotator in random order. Reliability between the first and second annotators was substantial, with Cohen's Kappa = 0.85 (*IQR* = 0.09, range = 0.51–0.97). A third annotator then coded only the frames where the first two annotators disagreed on the number of faces (*n* = 3,959; 3.9% of frames across both studies). A consensus was reached when 2 out of the 3 annotators coded the same number of faces present in each video frame. All images reached agreement between coders. Based on manual annotation, at least part of a face was visible in 39,874 frames in Study 1a (HMET 4mo; 91.3% of that study's total frames) and 13,966 in Study 1b (HMET 8mo; 23.8% of that study's total frames).

### 2.3 Study 2

#### 2.3.1 Participants

Data were collected from 10 typically developing children, aged 18–29 months (*Table* *2*). Nine of them were White‐British, and one was of mixed background. Seven participants were male, and three were female. Eligibility criteria included being from monolingual English‐speaking households (≥95% English), having no diagnosed neurodevelopmental conditions, and contributing at least 40 minutes of usable footage (two‐thirds of the 1‐hour target). Participants were recruited through existing databases and opportunity sampling, including via social media, leaflets in local nurseries, events and word‐of‐mouth. Ethical approval was obtained from Cardiff University School of Psychology Ethics Committee (EC.23.08.08.6821GRA). Informed consent was obtained from parents. Participants were given a small gift (e.g., a T‐shirt, a book) and a £10 multi‐retailer gift voucher in return for their participation. In addition, three participants who initially recorded less than the eligibility threshold of 40 minutes of duration were invited to provide an extra recording and received an additional £5 voucher.

**TABLE 2:** Demographic characteristics of Study 2 sample.

	Study 2 (Headcam 18–29mo)
Sex: Male/Female	7/3
Ethnicity:	
White	9
Black	0
Asian or Pacific Islander	0
More than one	1
Not reported	0
Highest level of parental education:	
Secondary education	1
A‐levels	0
Vocational/College	2
Undergraduate degree	1
Postgraduate degree	6
Total household income:	28,000–98,000
	*Mdn* = 68,500, *IQR* = 37,000

#### 2.3.2 Procedures

##### 2.3.2.1 Equipment

**FIGURE 2:**
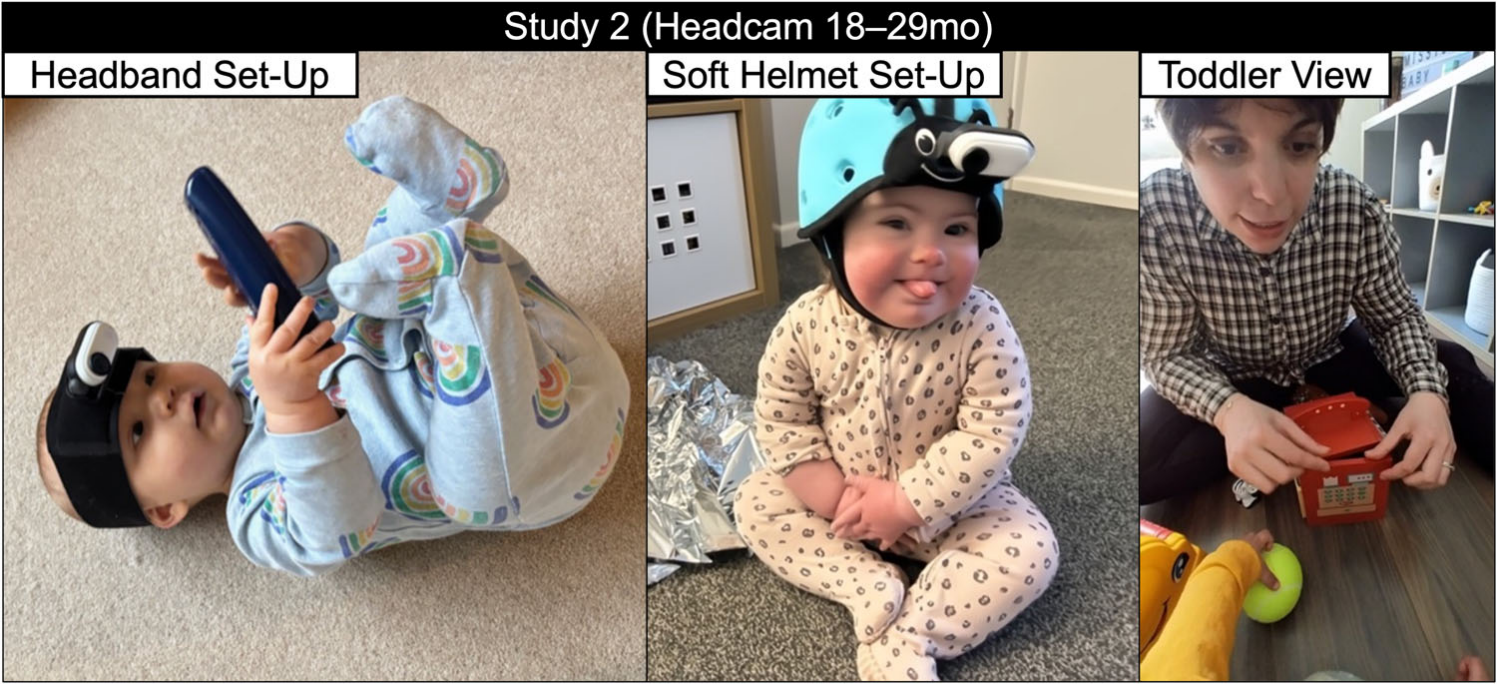
TinyExplorer headband (left) and TinyExplorer soft helmet (middle) configurations. Example video frame shown on the right.

A custom‐assembled headcam system (TinyExplorer gear; Nikolov et al. 2024; https://osf.io/95wvn/; see Figure 2) was used to record egocentric video data. The camera recorded at 50 frames per second (more individual frames within the same time period means that each frame captures a smaller portion of the movement, resulting in less blur). The analysed videos were vertically oriented (1080 × 1920 pixels; 9:16 aspect ratio) with a horizontal FOV of 80° and a vertical FOV of 116° (see Figure 2, right‐hand panel).

##### 2.3.2.2 Video Recordings

Two TinyExplorer headgears were mailed to families for home recording. Caregivers were asked to place the gear on their child's head and record during playtime for up to 1 hour in total (∼30 minutes per camera; ∼1 hour across two cameras). Instructions were provided on how to pause the camera if necessary. Families were asked to record at home on a typical day during playtime, when the child was ‘at their best’, and outside of meal and nap times. After the equipment was posted back, videos were exported and clipped to exclude segments with prolonged physical interference (e.g., touching or adjusting the camera or helmet for more than 5 seconds), significant misalignment of the camera view, or visible nudity beyond what would typically be seen at a public beach. Brief or minor physical interference that did not affect footage centrality was retained. Clipped sections accounted for a median of 19% of the total video duration per participant (*IQR* = 56%, range = 0%–82%).

##### 2.3.2.3 Manual Annotation of Faces in Child's First‐Person View

Frames were sampled at 1 Hz (one frame per second) for both manual and automated annotation, as previous research suggests that coarser sampling is sufficiently dense to capture major regularities in children's visual environments (Fausey et al. 2016). This resulted in a total of 34,013 frames across all children (*Mdn* = 3,503.5, *IQR* = 691.8, range = 1,996–4,176). Annotators completed a 1‐h training session where they were introduced to the custom programme for annotation and were trained on example data. They were not blind to participant identity, as participant IDs were visible at the start of the annotation session. Following Fausey et al. (2016), four human annotators labelled each image for the presence of at least one face (yes/no). We used a custom task designed in PsychoPy version 2023.2.3 (Peirce et al. 2019) which involved presenting one frame a time in the middle of the screen and asking annotators to indicate with a key press (key ‘A’ for absent and key ‘P’ for present) if the frame contained at least one part of a human face (e.g., a single eye visible was counted as a face). Annotation was completed in 1‐hour blocks, and the order of presentation of frames was randomised. Each human annotator spent approximately 2 hours annotating each dataset, with a total estimate of 80 hours for all 34,013 frames. Following Fausey et al. (2016), the criterion for assigning a label to each frame required 3 out of the 4 annotators to agree. Interrater reliability was evaluated using Fleiss’ kappa, with a median κ of 0.86 (*IQR* = 0.04; range = 0.78–0.92) across datasets, indicating a high level of agreement among raters. Based on manual annotation, 6,959 frames (20.5% of total frames) contained at least part of a face in Study 2.

### 2.4 Automated Annotation of Faces

We used the DeepFace library (Serengil and Özpinar 2024; for overview, see Table 3) in Python to apply 13 pre‐implemented face detection models to datasets from Study 1a (HMET 4mo), Study 1b (HMET 8mo) and Study 2 (Headcam 18–29mo). All models were run using their default detection thresholds. No additional training or tuning was applied.

**TABLE 3:** Overview of face detection algorithms from the DeepFace library.

Algorithm	Type	Default threshold (not based on developmental datasets)	Technical summary (not based on developmental datasets)
*Dlib* (King 2009)	Feature‐based	No configurable confidence threshold. Faces are detected if they pass the internal detection criteria.	A feature‐based face detector that works well for clear, front‐facing faces in good lighting. It is fast and runs efficiently on standard computers, but less accurate in complex or real‐world conditions.
*OpenCV* (Bradski 2000)	Feature‐based	No configurable confidence threshold. Faces are detected if they pass the cascade's internal criteria.	*OpenCV* uses a set of quick, step‐by‐step checks (called Haar cascades) to detect faces. Very fast but less accurate, especially on complex or varied faces.
*CenterFace* (Xu et al. 2020)	Deep learning	0.35	An anchor‐free model detecting faces and landmarks in a single shot. Balanced in accuracy and speed.
*FastMTCNN* (Timesler 2021; Zhang et al. 2016)	Deep learning	1. Generate candidate face regions (threshold = 0.60) 2. Filter and refine candidate regions (threshold = 0.70) 3. Final face detection and landmark localisation (threshold = 0.80)	Streamlined version of *MTCNN* designed for higher speed at some cost to accuracy.
*MediaPipe* (Lugaresi et al. 2019)	Deep learning	0.70	Optimised for smartphones, tablets, and the web. *MediaPipe* is fast and lightweight. Good trade‐off between accuracy and speed.
*MTCNN* (Zhang et al. 2016)	Deep learning	Same as *FastMTCNN* (above)	Three‐stage detector using convolutional neural networks for coarse‐to‐fine face localisation. Reasonably accurate, moderate speed.
*RetinaFace* (Deng et al. 2020)	Deep learning	0.90	An accurate detector that also localises facial landmarks. Slower but highly precise.
*SSD* (Liu et al. 2016)	Deep learning	0.90	Single‐shot detector that processes the image in one pass. Efficient and moderate in speed.
*YOLOFace v8 (N)/v11 (N)/v11 (S)/v11 (M)* (Akanametov 2024; Jocher et al. 2023)	Deep learning	0.25	Fast one‐stage object detectors using convolutional neural networks. Optimised for speed and real‐time applications.
*YuNet* (Wu et al. 2023)	Deep learning	0.90	Lightweight face detector using regression‐based methods. Suitable for real‐time tasks on smartphones, tablets, or low‐resource devices.

DeepFace face detection methods can be broadly classified into two categories based on their underlying approaches: *feature‐based* methods and *deep learning‐based* methods. *Feature‐based* methods form the early foundation of face detection. For instance, the *Viola–Jones* detector, popularised through *OpenCV* (Bradski 2000), was one of the first widely used face detection techniques, relying on simple image features and a step‐by‐step filtering process to quickly identify faces. Similarly, *Dlib* (King 2009) uses a method that looks for patterns in pixel intensity to detect facial structures. These approaches are computationally efficient and require minimal processing power compared to deep learning methods, making them suitable for real‐time monitoring or use on devices with limited hardware. Additionally, they are relatively easy to implement and do not require large training datasets. However, they struggle with more complex real‐world scenarios, such as detecting faces that are partially covered, at an angle, or in low‐quality images (Zafeiriou et al. 2015), which are challenges commonly encountered in dynamic children's views.

*Deep learning‐based* methods have transformed face detection by using artificial neural networks to recognise patterns in images. Unlike feature‐based methods that rely on manually designed rules, these models automatically learn to detect facial features from large datasets, improving accuracy and adaptability. One widely used approach, the Single Shot MultiBox Detector (*SSD*; Liu et al. 2016), quickly identifies faces in a single step, making it efficient for real‐time applications. Multi‐Task Cascaded Convolutional Neural Networks (*MTCNN*; Zhang et al. 2016) take this further by detecting faces while also pinpointing key facial features, such as eyes and noses, to improve precision. A more optimised version, *FastMTCNN* (Timesler 2021; Zhang et al. 2016), increases speed while maintaining accuracy. Other advanced deep learning models include *RetinaFace* (Deng et al. 2020), which predicts face locations and facial landmarks with high precision, and *MediaPipe* (Lugaresi et al. 2019), a lightweight model designed for smartphones and tablets that enables real‐time face tracking. More recent models, such as *YuNet* (Wu et al. 2023) and *CenterFace* (Xu et al. 2020) offer fast and efficient face detection while requiring less processing power. Finally, *YOLOFace* (Akanametov 2024; Jocher et al. 2023) is a high‐speed, single‐stage face detector built on the *YOLO* (You Only Look Once) architecture, the well‐established framework for real‐time object detection. Adapted specifically for face detection, *YOLOFace* fine‐tunes *YOLO*’s general object detection backbone to focus on facial features. Taken together, these deep learning‐based methods are likely to be particularly useful in developmental research because they can handle challenges such as changes in lighting, different head positions, and partial occlusions, which are common issues when studying infants and young children in natural environments.

### 2.5 Validation Plan

Frames in which at least one face was automatically detected were compared against the ground truth manual annotations. Because we were interested in overall face presence rather than individual face counts, we used a binary outcome of whether at least one face was detected or not, rather than considering multiple detected faces. In evaluating the performance of face detection algorithms, we used the *F*_1_ score per participant as the primary metric. The *F*_1_ score is the harmonic mean, providing a single measure that balances two important aspects: (1) *precision* (proportion of detected faces that were correct) and (2) *recall* (proportion of actual faces that were successfully detected). It is termed *F*_1_ because it represents the equal‐weight (*β* = 1) case of the more general F‐measure family, in which precision and recall are weighted equally. It is calculated by taking the product of precision and recall, multiplying by two, and then dividing by their sum. The *F*_1_ score, a common evaluation metric for algorithms (Chinchor 1992; Sokolova and Lapalme 2009), is also particularly useful in our study due to the imbalanced nature of egocentric video data, where instances of faces and non‐faces may vary significantly. The *F*_1_ score was prioritised over accuracy as the primary evaluation metric because it captures the balance between false positives and false negatives, whereas accuracy can inflate performance estimates in class‐imbalanced datasets. By considering both false positives and false negatives, the *F*_1_ score offers a more comprehensive evaluation of algorithm performance, ensuring neither over‐detection nor under‐detection is overlooked. This makes it a robust and reliable metric for assessing face detection algorithms in real‐world contexts.

## 3 Results

### 3.1 Algorithm Performance

**FIGURE 3:**
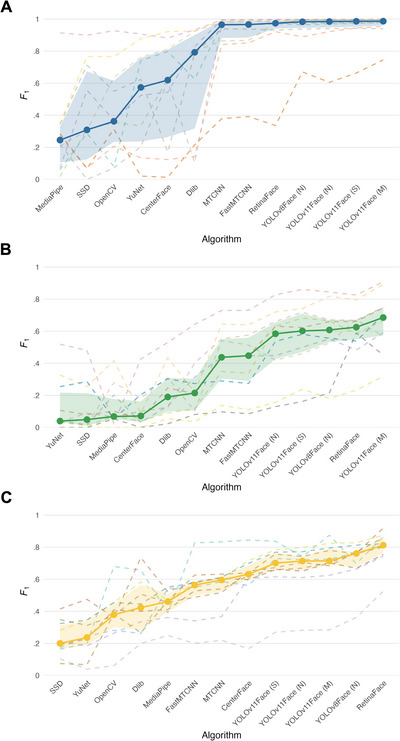
*F*_1_ score (harmonic mean of precision and recall) of face detection algorithms from the DeepFace Library on egocentric headcam videos across (A) Study 1a (HMET 4mo); (B) Study 1b (HMET 8mo); and (C) Study 2 (Headcam 18–29mo). Each dashed line corresponds to one participant; the solid line represents the median across participants. The shaded area indicates the interquartile range (IQR), which represents the middle 50% of values. Within each graph, models are ordered by ascending median *F*_1_.

Bayesian mixed‐effects beta regression models were employed to evaluate differences in *F*_1_ (see Table 4) scores across the 13 face detection algorithms across the three studies: Study 1a (HMET 4mo; Figure 3A), Study 1b (HMET 8mo; Figure 3B) and Study 2 (Headcam 18–29mo; Figure 3C). Each model incorporated the algorithm as a fixed effect and participant as a random intercept. Given that the *F*_1_ score is a continuous measure bounded between 0 and 1, a beta likelihood was used. Weakly informative priors were specified: a student‐*t* (3, 0, 2.5) prior was applied to the intercept, and group‐level standard deviations, flat (uniform) priors were assigned to the fixed effects, and the shape (precision) parameter ϕ was given a Gamma (0.01, 0.01) prior. To address boundary values of 0 and 1 in the *F*_1_ scores, the data were rescaled using a small offset (0.0001), ensuring that all values fell within the open interval (0, 1), which is required for beta regression (Smithson and Verkuilen 2006). The model was estimated using Markov Chain Monte Carlo (MCMC) sampling with four chains and 4,000 iterations per chain (2,000 warm‐up), implemented via the *brms* package in R (Bürkner 2017), leveraging the *CmdStanR* backend for efficient computation. The number of chains and iterations was selected to ensure stable posterior estimation and reliable convergence across model parameters.

**TABLE 4:** Summary of *F*_1_, Precision (P) and Recall (R) by algorithm and study.

	Study1a (HMET 4mo)			Study 1b (HMET 8mo)			Study 2 (Headcam 18–29mo)		
Algorithm	*F*_1_	*P*	*R*	*F*_1_	*P*	*R*	*F*_1_	*P*	*R*
*CenterFace*	0.62 (0.54)	1 (0)	0.45 (0.52)	0.07 (0.13)	1 (0.04)	0.04 (0.07)	0.63 (0.09)	0.69 (0.23)	0.72 (0.20)
*Dlib*	0.79 (0.58)	1 (0)	0.66 (0.61)	0.19 (0.21)	0.46 (0.38)	0.12 (0.19)	0.42 (0.28)	0.76 (0.12)	0.33 (0.19)
*FastMTCNN*	0.97 (0.10)	1 (0.01)	0.94 (0.18)	0.45 (0.27)	0.45 (0.46)	0.44 (0.21)	0.56 (0.09)	0.45 (0.14)	0.76 (0.17)
*MTCNN*	0.96 (0.11)	1 (0.01)	0.94 (0.19)	0.44 (0.24)	0.48 (0.47)	0.41 (0.21)	0.60 (0.09)	0.49 (0.16)	0.76 (0.18)
*MediaPipe*	0.25 (0.22)	1 (0.07)	0.14 (0.15)	0.07 (0.12)	0.29 (0.16)	0.05 (0.10)	0.46 (0.11)	0.63 (0.18)	0.35 (0.11)
*OpenCV*	0.36 (0.38)	0.99 (0.07)	0.23 (0.30)	0.21 (0.20)	0.22 (0.21)	0.21 (0.19)	0.38 (0.14)	0.32 (0.14)	0.48 (0.17)
*RetinaFace*	0.97 (0.05)	1 (0)	0.95 (0.09)	0.63 (0.12)	0.99 (0.04)	0.46 (0.22)	0.81 (0.10)	0.97 (0.15)	0.78 (0.17)
*SSD*	0.31 (0.55)	1 (0)	0.18 (0.44)	0.05 (0.20)	1 (0.25)	0.03 (0.12)	0.20 (0.15)	0.99 (0.02)	0.11 (0.10)
*YOLOv11Face (M)*	0.99 (0.04)	1 (0)	0.98 (0.07)	0.69 (0.18)	0.72 (0.42)	0.69 (0.20)	0.71 (0.10)	0.68 (0.23)	0.87 (0.15)
*YOLOv11Face (N)*	0.98 (0.05)	1 (0.01)	0.97 (0.09)	0.58 (0.23)	0.60 (0.35)	0.62 (0.19)	0.71 (0.09)	0.64 (0.21)	0.78 (0.17)
*YOLOv11Face (S)*	0.99 (0.04)	1 (0)	0.97 (0.08)	0.60 (0.24)	0.55 (0.33)	0.65 (0.16)	0.70 (0.11)	0.65 (0.22)	0.83 (0.14)
*YOLOv8Face (N)*	0.98 (0.05)	1 (0)	0.97 (0.10)	0.61 (0.14)	0.69 (0.35)	0.56 (0.23)	0.76 (0.09)	0.71 (0.21)	0.79 (0.19)
*YuNet*	0.57 (0.52)	1 (0)	0.40 (0.47)	0.04 (0.19)	1 (0)	0.02 (0.11)	0.24 (0.15)	1 (0.03)	0.13 (0.10)

*Note*: Values represent the median, with interquartile range (*IQR*) in parentheses. Algorithms are arranged alphabetically.

Leave‐one‐out information criterion (LOOIC) was used to assess whether algorithm inclusion meaningfully improved model performance. LOOIC provides an estimate of out‐of‐sample predictive accuracy based on approximate leave‐one‐out cross‐validation (Vehtari et al. 2017). Where necessary, moment matching was applied to stabilise estimates for influential observations (i.e., when Pareto‐*k* > 0.7). In Study 1a (HMET 4mo), the inclusion of the algorithm as a fixed effect significantly improved model fit, as evidenced by LOOIC (ΔLOOIC = 69.7, *SE* = 8.5). The model explained a large proportion of the variance in *F*_1_ scores (Bayesian *R* ^2^ = 0.83, 95% CI [0.78, 0.87]), with observed values ranging approximately from 0.00 to 1. In Study 1b (HMET 8mo), the full model also outperformed the null model (ΔLOOIC = 96.8, *SE* = 10.7), and explained a substantial proportion of variance in *F*_1_ scores (Bayesian *R* ^2^ = 0.88, 95% CI [0.85, 0.90]), with observed *F*_1_ scores ranging from 0.01 to 0.91. In Study 2 (Headcam 18–29mo), the model also demonstrated a significant improvement over the null model (ΔLOOIC = 104.8, *SE* = 9.0), with a high proportion of variance explained (Bayesian *R* ^2^ = 0.88, 95% CI [0.86, 0.90]). The observed *F*_1_ scores ranged from 0.04 to 0.92, indicating a wide spread of values similar to those observed in Study 1a (HMET 4mo) and Study 1b (HMET 8mo). This means that, across all studies, algorithm choice explained meaningful variation in detection performance.

Pairwise Wilcoxon rank‐sum tests with Bonferroni adjustment were conducted on participant‐level median *F*_1_ collapsed across algorithms to compare performance across studies. Results showed that Study 1a (HMET 4mo) had significantly higher *F*_1_ than both Study 1b (HMET 8mo), *W* = 93, *p*_adj_ = 0.001, and Study 2 (Headcam 18–29mo), *W* = 9, *p*_adj_ = 0.003. The comparison between median *F*_1_ in Study 1b (HMET 8mo) and Study 2 (Headcam 18–29mo) was not significant, *W* = 70, *p*_adj_ = 0.429. Overall, the findings confirm that algorithm choice significantly influences face detection performance, with consistent ranking patterns emerging across studies, and performance being strongest in Study 1a (HMET 4mo). Across all studies, several algorithms demonstrated consistently high performance, including *YOLOv11Face (M)*, *RetinaFace* and *YOLOv8Face (N)*, while others showed weaker or uncertain effects. The complete pairwise comparison results are provided in Table 5.

**TABLE 5:** Model comparisons: estimates with 95% confidence intervals (CI), estimate errors, evidence ratios (*BF*_10_) and posterior probabilities.

Study	Comparison	Estimate (95% CI)	Est. error	*BF*_10_	Post. prob
Study 1a (HMET 4mo)	*YOLOv11Face (M)* > *YOLOv11Face (S)*	0.10 (–0.58, 0.79)	0.42	1.49	0.60
	*YOLOv11Face (S)* > *YOLOv11Face (N)*	0.10 (–0.58, 0.80)	0.42	1.48	0.60
	*YOLOv11Face (N)* > *YOLOv8Face (N)*	0.00 (–0.70, 0.68)	0.42	1.00	0.50
	*YOLOv8Face (N)* > *RetinaFace*	0.26 (–0.41, 0.95)	0.42	2.84	0.74
	*RetinaFace* > *FastMTCNN*	0.17 (–0.49, 0.83)	0.40	2.04	0.67
	*FastMTCNN* > *MTCNN*	0.02 (–0.64, 0.66)	0.40	1.09	0.52
	***MTCNN* > *Dlib***	**1.73 (1.13, 2.34)**	**0.37**	**>1000**	**1**
	*Dlib* > *CenterFace*	0.30 (–0.75, 1.36)	0.64	2.15	0.68
	*CenterFace* > *YuNet*	0.23 (–0.84, 1.28)	0.64	1.76	0.64
	***YuNet* > *OpenCV***	**0.48 (–0.06, 1.02)**	**0.33**	**12.89**	**0.93**
	***OpenCV* > *SSD***	**0.31 (–0.22, 0.84)**	**0.32**	**5.10**	**0.84**
	***SSD* > *MediaPipe***	**0.58 (0.01, 1.14)**	**0.34**	**20.05**	**0.95**
Study 1b (HMET 8mo)	***YOLOv11Face (M)* > *RetinaFace***	**0.28 (–0.13, 0.70)**	**0.25**	**6.38**	**0.86**
	*RetinaFace* > *YOLOv8Face (N)*	0.15 (–0.26, 0.56)	0.25	2.59	0.72
	*YOLOv8Face (N)* > *YOLOv11Face (S)*	0.05 (–0.33, 0.44)	0.24	1.43	0.59
	***YOLOv11Face (S)* > *YOLOv11Face (N)***	**0.19 (–0.20, 0.57)**	**0.24**	**3.67**	**0.79**
	***YOLOv11Face (N)* > *FastMTCNN***	**0.45 (0.06, 0.85)**	**0.24**	**36.91**	**0.97**
	*FastMTCNN* > *MTCNN*	−0.02 (–0.42, 0.37)	0.24	0.85	0.46
	***MTCNN* > *OpenCV***	**0.94 (0.53, 1.36)**	**0.26**	**>1000**	**1**
	*OpenCV* > *Dlib*	0.09 (–0.35, 0.52)	0.27	1.71	0.63
	***Dlib* > *CenterFace***	**3.44 (2.46, 4.41)**	**0.60**	**>1000**	**1**
	*CenterFace* > *MediaPipe*	−2.31 (–3.30, –1.29)	0.64	0.00	0.00
	*MediaPipe* > *SSD*	−0.09 (–0.66, 0.48)	0.35	0.68	0.40
	*SSD* > *YuNet*	−0.16 (–0.69, 0.36)	0.32	0.44	0.31
Study 2 (Headcam 18–29mo)	***RetinaFace* > *YOLOv8Face (N)***	**0.49 (0.17, 0.81)**	**0.19**	**215.22**	**1**
	*YOLOv8Face (N)* > *YOLOv11Face (M)*	0.05 (–0.24, 0.35)	0.18	1.59	0.61
	***YOLOv11Face (M)* > *YOLOv11Face (N)***	**0.15 (–0.13, 0.43)**	**0.18**	**4.12**	**0.80**
	*YOLOv11Face (N)* > *YOLOv11Face (S)*	−0.03 (–0.32, 0.26)	0.17	0.76	0.43
	*YOLOv11Face (S)* > *CenterFace*	−0.16 (–0.73, 0.41)	0.35	0.45	0.31
	***CenterFace* > *MTCNN***	**0.70 (0.15, 1.27)**	**0.35**	**46.62**	**0.98**
	*MTCNN* > *FastMTCNN*	0.10 (–0.17, 0.36)	0.16	2.70	0.73
	***FastMTCNN* > *MediaPipe***	**0.39 (0.12, 0.65)**	**0.17**	**122.08**	**0.99**
	*MediaPipe* > *Dlib*	0.01 (–0.26, 0.28)	0.16	1.09	0.52
	***Dlib* > *OpenCV***	**0.29 (0.02, 0.56)**	**0.16**	**24.00**	**0.96**
	***OpenCV* > *YuNet***	**0.68 (0.39, 0.97)**	**0.17**	**>1000**	**1**
	*YuNet* > *SSD*	0.09 (–0.21, 0.40)	0.19	2.25	0.69

*Note*: For each study, algorithms were ordered by their median *F*_1_ score (highest to lowest), and adjacent algorithms were compared sequentially using directional hypotheses (e.g., A > B). Values in brackets represent the lower and upper bounds of the 95% confidence interval. Comparisons with *BF*_10_ > 3, indicating moderate evidence for a significant difference, are shown in bold.

### 3.2 Confidence Threshold Sensitivity

To further examine the top‐performing models, we investigated their confidence threshold sensitivity to better understand how detection performance varies across studies. The threshold for face detection models represents the confidence level required for an algorithm to classify a detected region as a face. For example, if the threshold is set at 0.50, the model will only label a detection as a face if it is at least 50% confident, filtering out lower‐confidence detections that may be false positives. This threshold is critical because it directly influences the balance between precision and recall. A lower threshold may result in higher recall, capturing more actual faces, but can also increase false alarms (detecting faces where there are none), which may reduce precision. Conversely, a higher threshold may reduce false alarms but increase missed detections by failing to register real faces with lower confidence scores.

**FIGURE 4:**
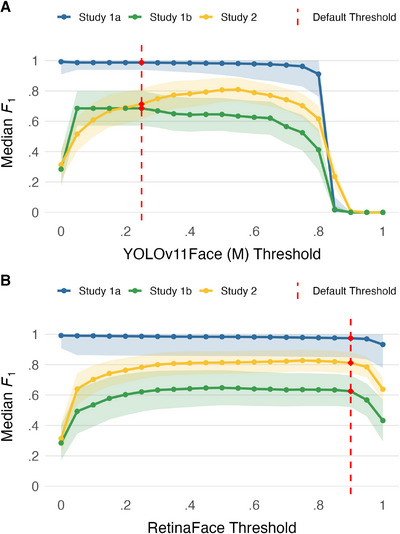
Median *F*_1_ Score across threshold values for (A) YOLOv11Face (M) and (B) RetinaFace face detection algorithms, illustrating performance variations in face detection. The shaded area represents the 95% confidence interval. The vertical red dashed line illustrates the default confidence threshold for each algorithm in the DeepFace Library.

As *RetinaFace* and *YOLO* variants consistently ranked among the top‐performing algorithms, we focused on *RetinaFace* and *YOLOv11Face (M)*, the newest and largest *YOLO* model implemented in the DeepFace library. We configured the detectors with a minimum confidence floor of 0.01, such that detections below this value were treated as false. For evaluation, we then calculated *F*_1_ scores at thresholds from 0.00 to 1 in increments of 0.05 (see Figure 4). Plateaus were defined as the range of thresholds whose median *F*_1_ was within 0.05 of the highest median *F*_1_ observed across thresholds for each algorithm and study, capturing regions of practically equivalent performance. We report only the plateau bounds to highlight the stability of performance rather than a single threshold. To derive overall plateaus across studies, we first averaged median *F*_1_ scores across all studies at each threshold. We then identified the highest median *F*_1_ observed across thresholds, and defined the overall plateau as the set of thresholds whose averaged *F*_1_ lay within 0.05 of this value.

When collapsed across studies, *RetinaFace* showed a broad plateau spanning 0.15 to 0.95. In Study 1a (HMET 4mo), *RetinaFace* exhibited a plateau from 0.01 to 0.95. Study 1b (HMET 8mo) showed a plateau extending from 0.20 to 0.90. In Study 2 (Headcam 18–29mo), the plateau ranged from 0.25 to 0.95.

Across studies, *YOLOv11Face (M)* showed a plateau spanning 0.15 to 0.65. In Study 1a (HMET 4mo), the plateau ranged from 0.01 to 0.75. Study 1b (HMET 8mo) showed a plateau from 0.05 to 0.50, and Study 2 (Headcam 18–29mo) had a plateau spanning 0.35–0.65.

Overall, *RetinaFace* generally exhibited broader plateau ranges, suggesting greater robustness to threshold selection, whereas *YOLOv11Face (M)* tended to show narrower plateaus, particularly in Study 1b (HMET 8mo) and Study 2 (Headcam 18–29mo), indicating greater sensitivity to threshold choice. In the context of egocentric recordings from children, for example, stricter confidence thresholds may be preferable to reduce false detections from toys and clutter, whereas lower thresholds may be more appropriate if the goal is to maximise the number of face detections. Thus, the most suitable threshold depends on the specific aims and constraints of the research context. Notably, the default thresholds used in DeepFace (0.90 for *RetinaFace* and 0.25 for *YOLOv11Face (M)*) both fell within the overall plateaus we identified, supporting their general suitability for developmental egocentric datasets.

### 3.3 Face Availability and Consistency of Individual Differences

To characterise developmental patterns, we first examined the proportion of frames containing faces based on manual annotation across the three studies: Study 1a (HMET 4mo; *Mdn* = 0.98, *IQR* = 0.06, *n* = 10), Study 1b (HMET 8mo; *Mdn* = 0.17, *IQR* = 0.16, *n* = 10) and Study 2 (Headcam 18–29mo; *Mdn* = 0.19, *IQR* = 0.07, *n* = 10) using pairwise Wilcoxon rank–sum tests with Bonferroni correction. Study 1a (HMET 4mo) showed a significantly higher proportion than Study 1b (HMET 8mo), *W* = 99, *p*_adj_ = 0.001, and also higher than Study 2 (Headcam 18–29mo), *W* = 99, *p*_adj_ = 0.001. No difference was observed between Study 1b (HMET 8mo) and Study 2 (Headcam 18–29mo), *W* = 46, *p*_adj_ = 1. These results indicate that faces were far more frequently present in the visual environments of 4‐month‐olds than in those of older infants and toddlers. Automated face detection with *RetinaFace* and *YOLOv11Face (M)* replicated this inferential pattern. Given the modest sample sizes, these findings should be interpreted cautiously.

**FIGURE 5:**
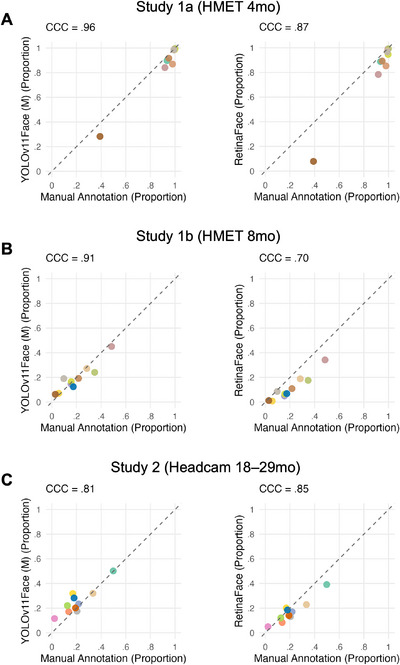
Scatterplots showing agreement between manual annotation and automated face detection estimates for (A) Study 1a (HMET 4mo), (B) Study 1b (HMET 8mo) and (C) Study 2 (Headcam 18–29mo) for *YOLOv11Face (M)* and *RetinaFace*. Each point represents the proportion of frames containing faces for an individual participant, plotted for a given algorithm (y‐axis) against the manually annotated ground truth (x‐axis). The dashed diagonal line indicates perfect agreement. Concordance correlation coefficient (CCC) values are shown to quantify overall agreement. The lowest‐scoring participant in Panel A was seated in a bumbo seat, leading to a downward head angle and reduced face visibility.

Building on this developmental pattern, we next asked whether automated detection preserved individual differences across participants. While *F*_1_ informs us about overall accuracy in balancing precision and recall, it does not capture whether algorithms preserve individual differences across participants. To address this, we evaluated the agreement between manual annotation and the two top‐performing automated face detection algorithms, *RetinaFace* and *YOLOv11Face (M)*, selected as the highest‐capacity *YOLO* variant implemented in the DeepFace library, by calculating the proportion of frames containing faces for each participant within each study: Study 1a (HMET 4mo), Study 1b (HMET 8mo) and Study 2 (Headcam 18–29mo). The concordance correlation coefficient (CCC) was used as a robust metric to assess the agreement between human annotation (treated as the gold standard) and algorithmic output. In addition, mean absolute error (MAE), mean signed error (bias) and Spearman's rank correlation (*r_s_*) were computed to provide a comprehensive picture of algorithm performance. In Study 1a (HMET 4mo; Figure 5A), *YOLOv11Face (M)* demonstrated very high agreement with manual annotation, with CCC = 0.96, MAE = 0.04, and a small negative bias (−0.04). Spearman's rank correlation also indicated strong rank‐order consistency, *r_s_* = 0.95, *p* < 0.001. *RetinaFace* also showed good performance in Study 1a (HMET 4mo), with CCC = 0.87, MAE = 0.08, and a bias of −0.08, along with strong rank‐order agreement, *r*_s_ = 0.88, *p* = 0.001. In Study 1b (HMET 8mo; Figure 5B), overall agreement was similarly high to that of Study 1a. *YOLOv11Face (M)* showed CCC = 0.91, MAE = 0.04, and a minimal bias of −0.01. Rank‐order consistency remained high, *r_s_* = 0.88, *p* = 0.001. *RetinaFace* showed CCC = 0.70, MAE = 0.09, and a negative bias of −0.09, with a strong rank‐order correlation, *r_s_* = 0.90, *p* < 0.001. In Study 2 (Headcam 18–29mo; Figure 5C), *RetinaFace* continued to show relatively strong agreement with manual annotation, CCC = 0.85, MAE = 0.05, and a bias of −0.04. The rank‐order correlation was also strong, *r_s_* = 0.78, *p* = 0.008. *YOLOv11Face (M)* showed CCC = 0.81, MAE = 0.05, and a positive bias of 0.05. Rank‐order consistency was moderate, *r_s_* = 0.67, *p* = 0.033.

Paired Wilcoxon signed‐rank tests with Bonferroni adjustment were used to compare participant‐level errors between algorithms within each study. For absolute error, *YOLOv11Face (M)* yielded significantly lower error than *RetinaFace* in Study 1a (HMET 4mo), *V* = 0, *p*_adj_ = 0.018, but not in Study 1b (HMET 8mo), *V* = 6, *p*_adj_ = 0.097, or in Study 2 (Headcam 18–29mo), *V* = 29, *p*_adj_ = 1. We also compared bias, finding that across all studies, *YOLOv11Face (M)* had consistently less negative bias than *RetinaFace* (all *V*s = 55, all *p*_adj_s = 0.018). Pairwise Wilcoxon rank‐sum tests with Bonferroni adjustment were then used to examine differences in absolute error and bias across studies separately for *RetinaFace* and *YOLOv11Face (M)*. All comparisons were nonsignificant (all *p*_adj _> 0.130), except for *YOLOv11Face (M)*, which showed a significantly more positive bias in Study 2 (Headcam 18–29mo) compared to Study 1a (HMET 4mo), *W* = 90, *p*_adj_ = 0.005.

Overall, manual annotation showed that faces were far more frequent in 4‐month‐olds’ visual environments than at later ages, and both algorithms demonstrated strong individual differences agreement with manual annotations across studies.

### 3.4 Robustness Checks

As participants contributed different numbers of frames, we assessed whether frame count influenced performance. Both weighted and unweighted median *F*_1_ scores are shown in Figure S1. Spearman's rank correlations between frame count and *F*_1_ showed no associations for any algorithm across studies (all *r*_s_s < 0.62, all *p*s > 0.050), except in Study 1b (HMET 8mo) for *MTCNN* (*r*_s_ = 0.77, *p* = 0.014) and *FastMTCNN* (*r*_s_ = 0.75, *p* = 0.018). Weighted and unweighted results showed convergent patterns, suggesting that frame count did not systematically bias outcomes. We also examined the effect of frame sampling rate on algorithm performance by comparing *F*_1_ scores across multiple downsampling levels (15, 7.5 and 1 Hz for Studies 1a and 1b; 1, 0.5 and 0.2 Hz for Study 2). Algorithm ranking and relative performance patterns were broadly consistent across sampling rates (Figure S2).

Given that face detection algorithms may be subject to racial and ethnic biases, we conducted a post hoc analysis on Study 1, which had the greatest variability in participant demographics. Algorithm performance, indexed by *F*_1_ score, did not differ significantly by race (all *p*s > 0.060) or ethnicity (all *p*s > 0.220) for any of the algorithms. These findings indicate that race and ethnicity were not significant factors for algorithm performance in this dataset, although this conclusion should be interpreted cautiously given the low number of participants.

## 4 Introducing the *TinyExplorer Detection App*

**FIGURE 6:**
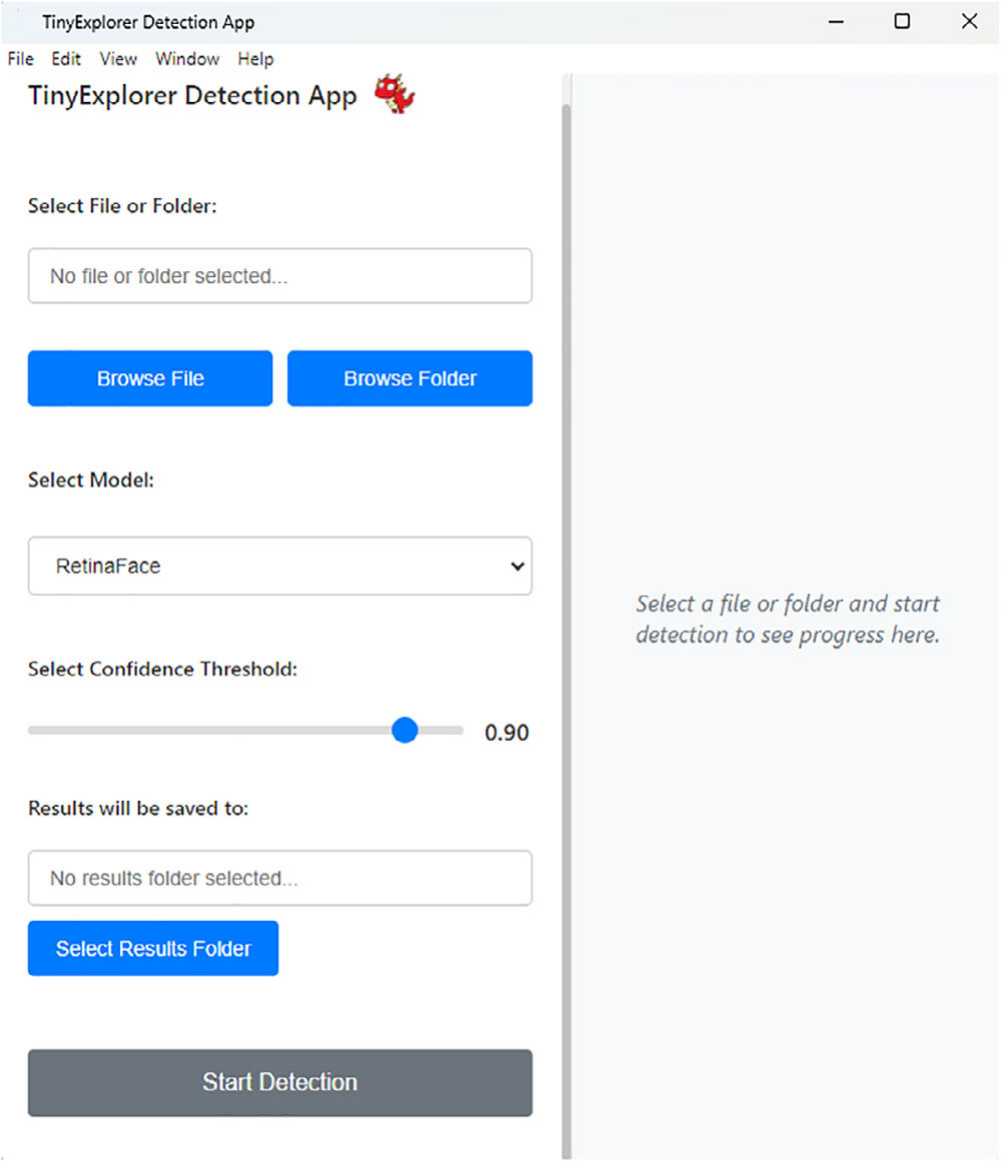
*TinyExplorer Detection App*, a user‐friendly graphical interface. This app integrates state‐of‐the‐art open‐source face detection algorithms into an easy‐to‐use software package, streamlining the process of analysing facial data in developmental research (Cardiff Babylab 2025).

For accessibility and privacy, we developed the *TinyExplorer Detection App* (see Figure 6; Cardiff Babylab 2025; https://cardiff‐babylab.github.io/tinyexplorer‐detection‐app), a user‐friendly local desktop application that provides users with direct access to state‐of‐the‐art face detection algorithms without requiring programming expertise. The application integrates multiple open‐source face detection models, including *YOLOv11 (M)* and *RetinaFace*, each optimised for different computational constraints and accuracy requirements. Researchers can upload videos or images, adjust detection thresholds, and review both summary statistics (e.g., total detected faces, average confidence scores) and detailed frame‐by‐frame detection logs. Data can be exported in widely compatible formats, and the app's modular architecture allows advanced users to swap out or update models with minimal code adjustments. Users can select from lightweight models suitable for real‐time processing to high‐accuracy models designed for offline batch processing, allowing researchers to balance detection precision with available computational resources. All models in our app run on standard laptops without requiring a discrete GPU. As an illustration of performance, benchmarking on a MacBook Pro (Apple M1 Max, 32 GB; macOS 14) using a random sample of 5,000 Caltech‐101 images (Li et al. 2022) yielded 11.17 images per second for *YOLOv11Face (M)* and 1.12 images per second for *RetinaFace*.

## 5 Discussion

In this study, we sought to reduce two key barriers that have limited the uptake of automated face detection in developmental research. The first is the rapidly evolving and technically complex landscape of available algorithms, which can make it difficult for researchers to identify and implement appropriate tools. The second barrier concerns accessibility and privacy, particularly when working with sensitive egocentric video data from children.

### 5.1 First Barrier: Algorithm Selection

We systematically evaluated the performance of 13 state‐of‐the‐art face detection algorithms from the DeepFace Library (*CenterFace*, *Dlib*, *FastMTCNN*, *MediaPipe*, *MTCNN*, *OpenCV*, *RetinaFace*, *SSD*, four *YOLOFace* versions and *YuNet*) on the first‐person visual scenes of children under 3 years of age. By evaluating these algorithms against manually annotated data from structured HMET recordings (Study 1a HMET 4mo, Study 1b HMET 8mo) and naturalistic home‐based headcam recordings (Study 2 Headcam 18–29mo), we assessed how well each aligned with this gold standard. We then compared these performance metrics across algorithms to identify the most effective methods for automated face annotation.

First, we found that algorithm performance varied substantially within each study. Although the models were trained on large, publicly available datasets such as WIDER FACE (Yang et al. 2016), which primarily contain third‐person images, they were not specifically trained on infant and toddler egocentric views. As a result, they may struggle with the unique visual characteristics of head‐mounted footage, including motion blur, partially visible or occluded faces, and unusual viewing angles. Indeed, many of the missed detections and false positives involved such challenging instances (see Figures S3–S5). One might assume that the type of recording device and video quality would be the primary drivers of these errors. However, our findings suggest a more nuanced picture. In Study 1a (HMET 4mo), several algorithms achieved similarly high median *F*_1_, scores descriptively led by *YOLOv11Face (M)*, followed by *YOLOv11Face (S)*, *YOLOv11Face (N)*, *YOLOv8Face (N)*, *RetinaFace*, *FastMTCNN* and *MTCNN*, with no statistically significant differences between models. The high concordance among these top models suggests that multiple modern detectors are viable for semi‐structured infant recordings. In Study 1b (HMET 8mo), *YOLOv11Face (M)* achieved the highest median *F*_1_ score and showed moderate evidence of outperforming *RetinaFace*. Other leading models, including *YOLOv8Face (N)* and *YOLOv11Face (S)*, exhibited comparable performance to *RetinaFace*, with no clear evidence of reliable differences between them. In Study 2 (Headcam 18–29mo), *RetinaFace* led in overall performance, followed closely by *YOLOv8Face (N)*, *YOLOv11Face (M)*, and *YOLOv11Face (N)*. Although differences among the top models were relatively small, *RetinaFace* appeared strongest in this naturalistic setting with toddlers. This aligns with previous research demonstrating its robustness in detecting partially occluded, non‐frontal faces under diverse lighting and scale conditions (Batagelj et al. 2021). Together, these findings show that while algorithm performance varies across developmental contexts and ages, *YOLOv11Face (M)* and *RetinaFace* were consistently among the stronger performers. *YOLOv11Face (M)* tended to perform well in the more structured settings with younger infants, where faces were often clearer and less occluded. *RetinaFace* appeared to perform relatively better in toddler headcam footage. We caution, however, that these are posthoc observations which need to be confirmed in future studies.

Algorithm performance was highest overall for Study 1a (HMET 4mo), in which parents were instructed to engage in toy play with their infants. Because the 4‐month‐old infants were unable to locomote or reach for objects independently, it is likely that parents led the interaction by positioning themselves in the centre of the infant's field of view and presenting toys in ways that minimised occlusion. This likely created stable visual scenes with unobstructed views of faces, supporting higher detection rates. Indeed, this pattern aligns with the importance of early attention to and processing of faces (Frank et al. 2009; Grossmann and Johnson 2007; Johnson 2005). In contrast, infants in Study 1b (HMET 8mo) were sitting freely, lying on their stomachs or crawling around the play space. Their increasing mobility and shifting posture likely resulted in more frequent occlusions and downward‐facing views, thereby reducing the frequency and visibility of faces captured in their egocentric view (Franchak et al. 2017; Long, Sanchez, et al. 2022). Moreover, the reduced algorithm performance for detecting faces in Study 1b (HMET 8mo) might be due to a change in the centrality of faces. Toddlers in Study 2 (Headcam 18–29month) showed a broadly similar level of algorithm performance to infants in Study 1b. Although parents were instructed to record during playtime, face proportions suggest that faces were less consistently present in toddlers’ egocentric views, likely reflecting their greater autonomy at this age. However, without finer‐grained behavioural measures, these interpretations should be treated with caution. Taken together, these findings reveal clear age‐related and contextual differences in automated face detection performance. These findings emphasise that automated face detection accuracy in developmental research is shaped not only by algorithmic design but also by the child's developmental stage and the nature of their interactions. This underscores the importance of context‐robust benchmarking when applying machine learning to real‐world child behaviour. In line with this, our results suggest that both *YOLOv11Face (M)* and *RetinaFace* offer strong, adaptable foundations for automated face detection in developmental research.

Secondly, we investigated the sensitivity of the two top‐performing algorithms (*YOLOv11Face (M)* and *RetinaFace*) to detection threshold settings, which define the minimum confidence score required to consider a face detection valid. This parameter is particularly important when applying pre‐trained models to new domains such as egocentric infant footage, as inappropriate thresholds can lead to excessive false positives or missed detections. Our results indicated that *RetinaFace* achieved high performance with plateau ranges extending broadly (e.g., 0.15–0.95 overall), indicating robustness to threshold selection across studies. In contrast, *YOLOv11Face (M)* showed narrower plateaus (e.g., 0.15–0.65 overall), particularly in Study 1b (HMET 8mo) and Study 2 (Headcam 18–29mo), where performance was more sensitive to small changes in threshold. Importantly, the default thresholds commonly applied in DeepFace (0.90 for *RetinaFace* and 0.25 for *YOLOv11Face (M)*) both fell within the identified plateaus, confirming their general appropriateness for use in developmental egocentric datasets. However, our analyses also suggested that higher thresholds (e.g., 0.55 in Study 2 for *YOLOv11Face (M)*) may further improve precision by reducing false positives, which are especially problematic when children's visual scenes contain frequent non‐social stimuli such as toys or background clutter.

Finally, we examined the extent to which *YOLOv11Face (M)* and *RetinaFace* preserved individual differences across participants by comparing their estimates with manual annotation. Across all studies, both *YOLOv11Face (M)* and *RetinaFace* showed strong correspondence with manual coding, indicating that participant‐level variation in face availability was captured by automated detection. In Study 1a (HMET 4mo), both algorithms exhibited a tendency to underestimate face presence relative to manual annotation, while still preserving the relative ordering of participants. In Study 1b (HMET 8mo), *YOLOv11Face (M)* showed minimal systematic bias relative to manual annotation, whereas *RetinaFace* tended to underestimate face presence. Despite these tendencies, participant‐level ordering remained largely consistent with manual annotation for both algorithms. In Study 2 (Headcam 18–29mo), *RetinaFace* showed a slight tendency towards underestimation, whereas *YOLOv11Face (M)* tended to overestimate face presence relative to manual coding. Importantly, both algorithms captured meaningful individual differences across participants. Given this preserved correspondence with manual annotation, model selection in developmental research need not be based on detection accuracy alone but may also be guided by practical and analytical considerations, including computational efficiency and the availability of auxiliary outputs. In this context, *YOLOv11Face (M)*, as the most recent and efficient *YOLO* variant, provides a lightweight and fast solution ideal for large‐scale annotation tasks. *RetinaFace*, while slower, delivers additional facial feature localisation, which may be beneficial for analyses focused on head orientation or interpersonal distance. Together, these findings support the construct validity of automated face detection for generating behaviourally meaningful data and suggest that such methods can capture not only overall accuracy but also finer‐grained behavioural patterns typically derived from manual annotation.

### 5.2 Second Barrier: Technical Implementation

The second barrier we addressed in this paper concerns accessibility and privacy. We developed the *TinyExplorer Detection App* (see Figure 6; Cardiff Babylab 2025; https://cardiff‐babylab.github.io/tinyexplorer‐detection‐app), a local‐processing desktop application that promotes efficiency, scalability and innovation in developmental science by democratising access to machine learning tools. The interface is designed for non‐experts, reducing technical barriers to adoption. Crucially, local processing ensures that footage remains on the researcher's machine, avoiding external transfer or cloud storage and helping to protect participant confidentiality. Together, these contributions lower both technical and ethical barriers, helping to make automated face detection a more practical and accessible tool for developmental science.

### 5.3 Key Limitations

There are two main limitations of the current study to be considered. The first one relates to the definition and reliability of ground truth annotation. Because our human annotators labelled a face as present when any part of a human face was visible, discrepancies between human annotation and automated detection may reflect differences in what individual algorithms consider sufficient evidence for a face rather than errors per se. Additionally, although each frame was annotated by multiple annotators in a randomised order, manual detection may still have been affected by annotator fatigue and subjective bias, and the fact that annotators were not blinded to participant ID number or timepoint. Further, toys and other household objects were sometimes visible in the home environment, including objects with face‐like features such as dolls. These objects were not manually annotated as faces, as ground‐truth coding was restricted to human faces only. The presence of face‐like objects may therefore introduce ambiguity when evaluating automated face detections in naturalistic settings. Future work should explicitly investigate how such objects influence automated face detection performance in everyday environments.

The second limitation pertains to algorithmic bias in face detection models. Automated face detectors often show uneven performance across demographic characteristics (e.g., skin tone, gender and age), with disparities linked to dataset composition and model design (Khalil et al. 2020; Menezes et al. 2021). Publications on the algorithms within DeepFace lack transparency about the datasets, making it impossible to assess their demographic representativeness and therefore inherent bias. In our egocentric videos, domain‐specific factors such as oblique viewpoints, occlusion, low or variable indoor lighting, motion blur and rapid scale changes further reduce accuracy and may compound demographic effects (e.g., darker skin in low light). Although these issues plausibly apply to our setting, we did not observe evidence of such bias in our dataset. However, this null finding should be interpreted cautiously, given the relatively small and demographically homogeneous sample (particularly Study 2, which included mostly White‐British families), limiting generalisability.

### 5.4 Future Directions

The current study focused on benchmarking existing automated face detection algorithms on infant/toddler egocentric video data, providing a stepping stone towards more scalable and reproducible analyses of children's everyday visual experiences. Future directions include integrating face detection outputs with gaze estimation data, particularly from head‐mounted eye‐trackers. Intersecting face bounding boxes with gaze vectors could offer detailed insights into infants’ visual attention, social referencing and learning opportunities in naturalistic environments. Another critical avenue is model fine‐tuning using context‐diverse data, extending beyond home settings to nurseries, playgrounds and broader demographic groups. Future work could pursue this investigation by incorporating algorithms trained on datasets designed to reduce bias, such as FairFace (Karkkainen and Joo 2021), BalancedFace (Mekonnen 2023) or Diversity in Faces (Merler et al. 2019). Evaluating the *TinyExplorer Detection App* itself will also be an important next step, allowing us to assess whether the tool effectively reduces technical and ethical barriers as envisaged.

## 6 Conclusions

By offering both concrete performance data and the *TinyExplorer Detection App*, we aim to empower developmental scientists to embrace these technologies, fostering more comprehensive and data‐rich explorations of early development. Our results confirm that while automated detection is not flawless, it is sufficiently accurate to significantly streamline research workflows. Ultimately, integrating automated face detection into standard developmental methodologies will open new opportunities to investigate children's real‐world social experiences on a scale and depth that manual annotation alone could not realistically achieve.

## Funding

This work was supported by a James S. McDonnell Foundation (JSMF) Opportunity Award (https://doi.org/10.37717/2022‐3711) and UKRI Future Leaders Fellowship (MR/X032922/1) awarded to HD, and by a James S. McDonnell Foundation (JSMF) Opportunity Award (https://doi.org/10.37717/2021‐3177) and by funding from the National Institutes of Health (K23MH120476) awarded to JB. Additional support was provided by National Institutes of Health funding (F32MH138129) awarded to JYH.

## Ethics Statement

The study was approved by the relevant departmental ethics committees and was conducted in accordance with the Declaration of Helsinki.

## Conflicts of Interest

The authors declare no conflict of interest.

## Supporting information




**Supporting File 1**: desc70148‐sup‐0001‐SuppInfo.docx

## Data Availability

The analysis scripts are available on OSF (https://doi.org/10.17605/osf.io/n49wp). The study data are not publicly available due to ethical and privacy restrictions. Access may be considered upon reasonable request via babylab@cardiff.ac.uk, subject to appropriate ethical approval and a data sharing agreement.
