# Supplementary material for: Making Machine Learning Accessible for Developmental Science: The Case of Automated Face Detection
Source: Dev Sci. 2026 Apr 20;29:e70148. doi: 10.1111/desc.70148 (PMC13095670; doi:10.1111/desc.70148)
Supplement: Supplementary file 1 — Supporting File 1: desc70148‐sup‐0001‐SuppInfo.docx [file DESC-29-e70148-s001.docx]

**Supporting Information**

**
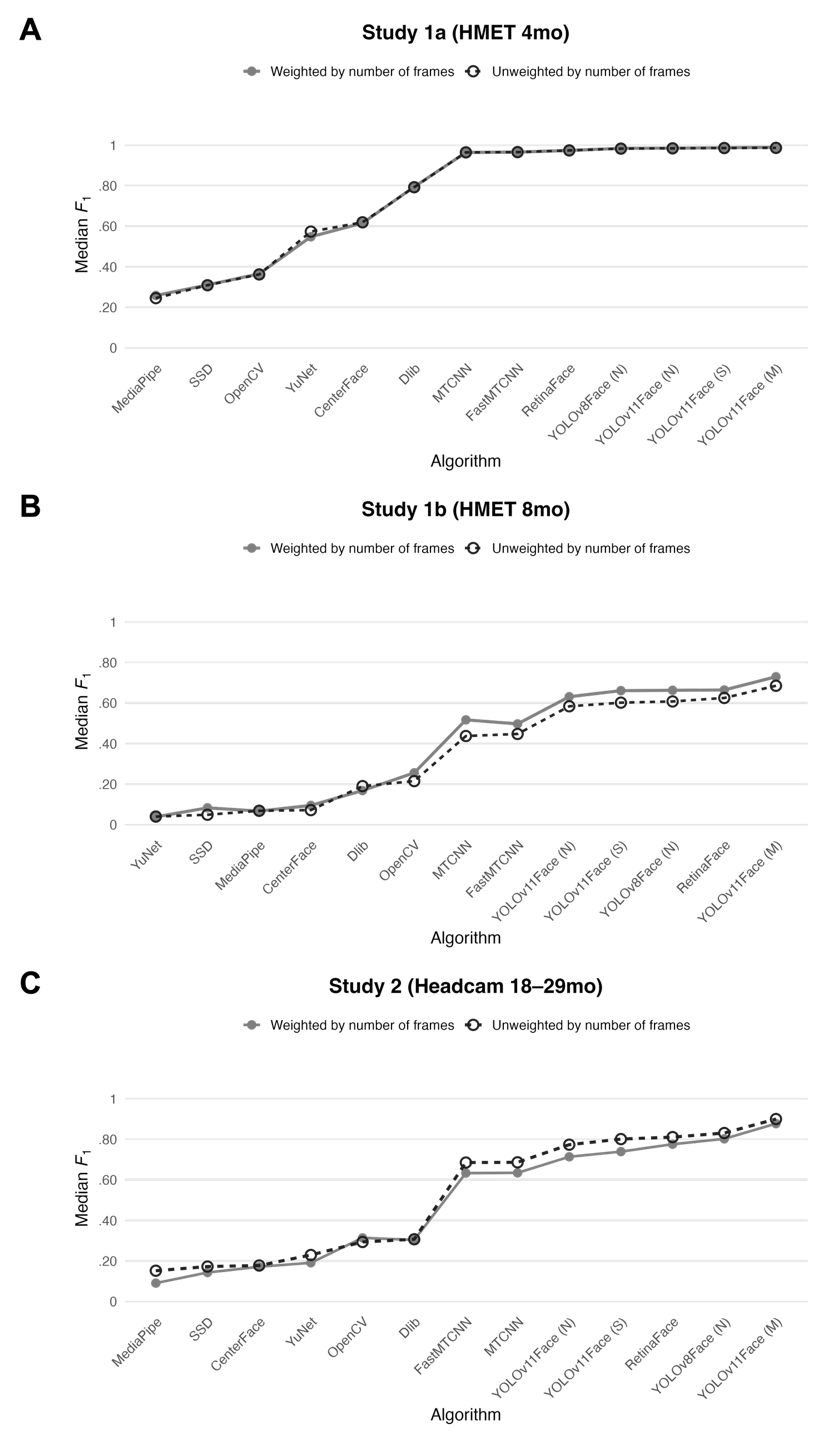
**

**Figure S1**

Unweighted vs. weighted *F*_1_ by algorithm across studies. (A) Study 1a (HMET 4mo), (B) Study 1b (HMET 8mo) and (C) Study 2 (Headcam 18–29mo). For each algorithm, the dashed black line and points show the unweighted median *F*_1_ across participants and the solid dark-grey line and points show the weighted median *F*_1_ (participant *F*_1_ values weighted by each participant’s frame count). Algorithms within each panel are ordered by ascending unweighted median *F*_1_.

**
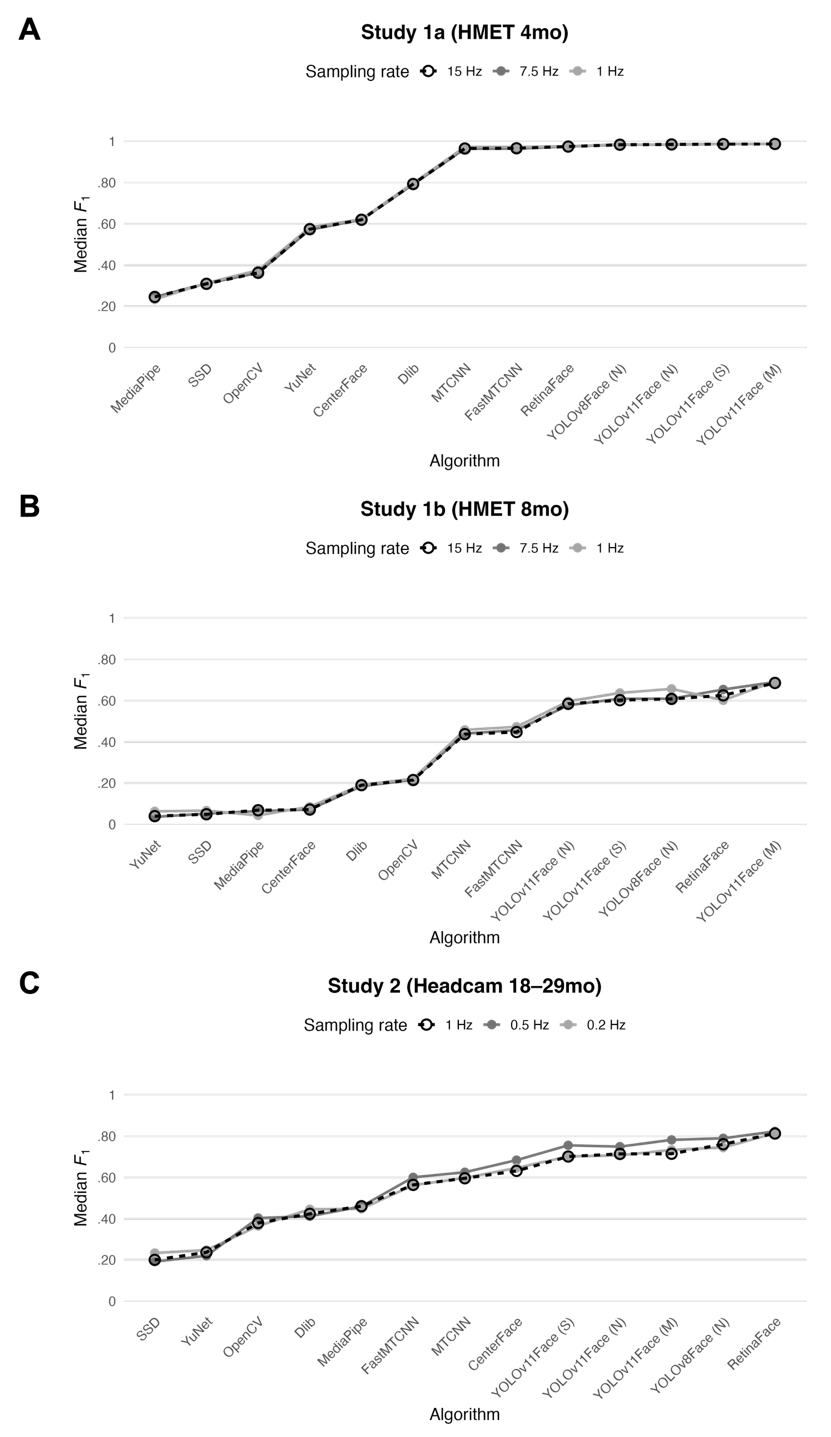
**

**Figure S2**

Median *F*_1_ by algorithm across sampling rates for (A) Study 1a (HMET 4mo), (B) Study 1b (HMET 8mo) and (C) Study 2 (Headcam 18–29mo). Algorithms within each panel are ordered by ascending median *F*_1_.

Study 1a (HMET 4mo)


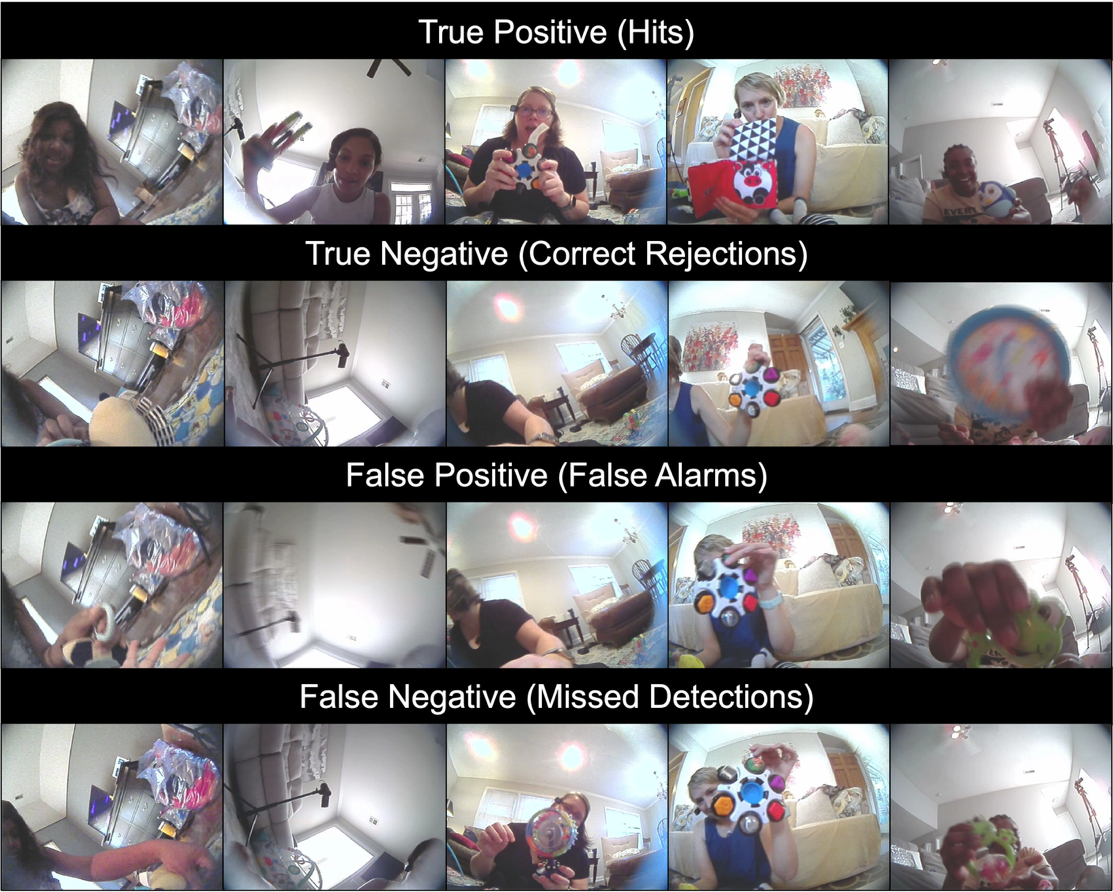


**Figure S3**

Example hits/correct rejections/false alarms/missed detections randomly sampled from the Study 1a (HMET 4mo) dataset.

Study 1b (HMET 8mo)


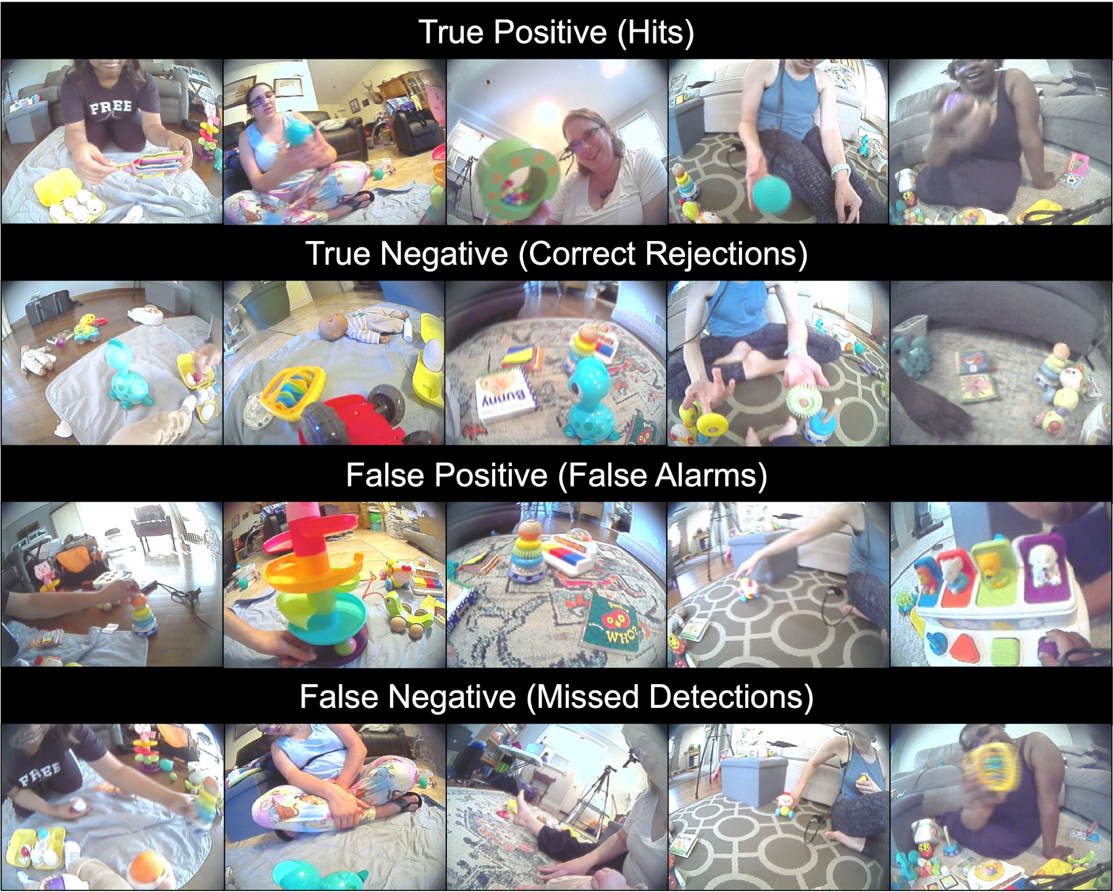


**Figure S4**

Example hits/correct rejections/false alarms/missed detections randomly sampled from the Study 1b (HMET 8mo) dataset.


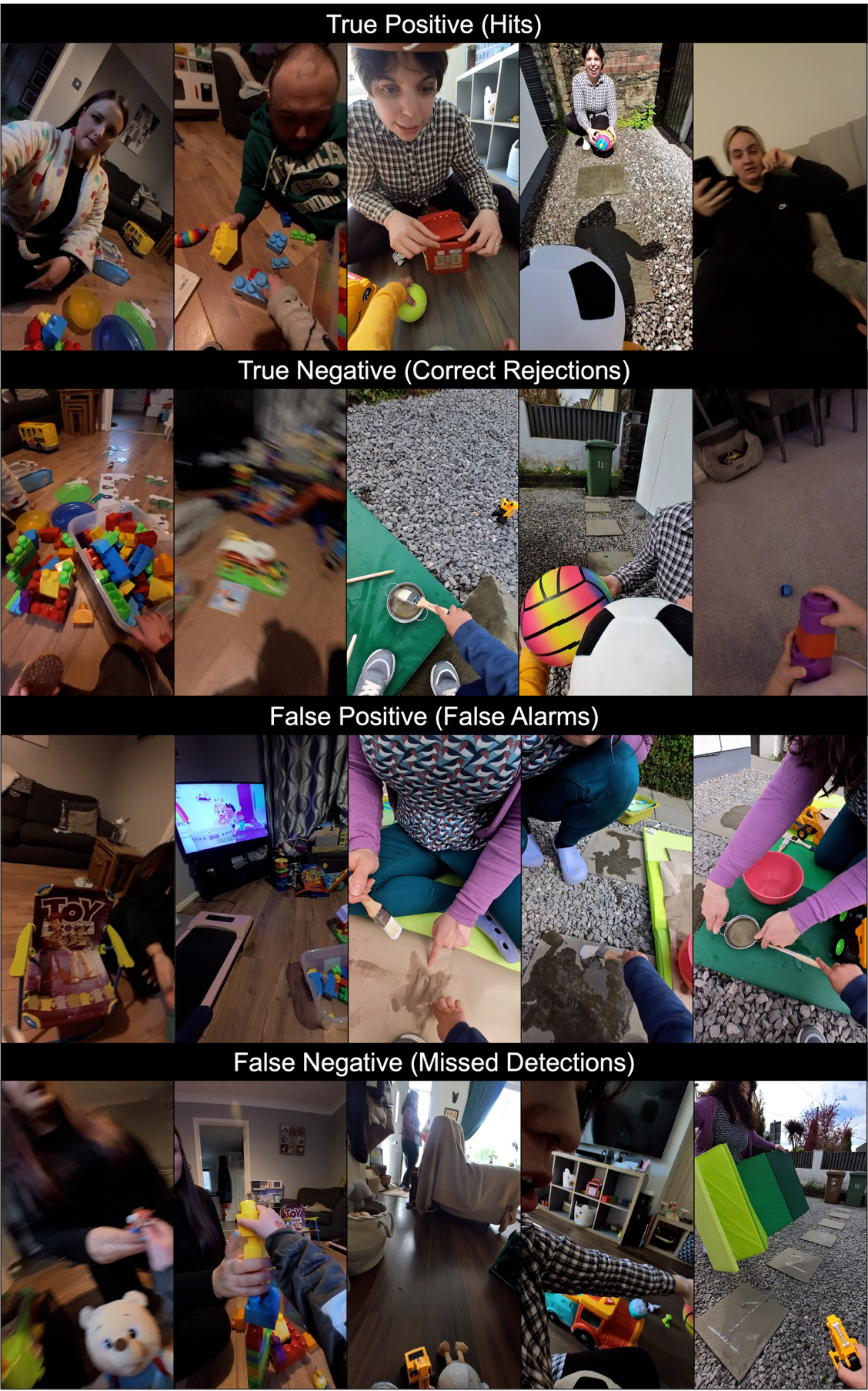


**Figure S5**

Example hits/correct rejections/false alarms/missed detections randomly sampled from the Study 2 (Headcam 18-29mo) dataset.
